# Influence of Driving Experience on Visual Search, Attentional Cue Utilization, and Inhibitory Control: Implications for Driving Performance

**DOI:** 10.3390/jemr19050110

**Published:** 2026-10-01

**Authors:** Prem Sudhakar Lawrence, Aiswaryah Radhakrishnan

**Affiliations:** Department of Optometry, SRM Medical College Hospital and Research Centre, Faculty of Medical and Health Science, SRM Institute of Science and Technology, Kattankulathur 603203, Tamil Nadu, India; premsudl@srmist.edu.in

**Keywords:** driving, visual search, cue, stop signal, cognitive skills

## Abstract

Background: Driving requires the integration of visual search, attentional processing, cue, and response inhibition. Driving experience and regular driving exposure may influence these cognitive processes, but their relative effects across specific cognitive performance remain unclear. Methods: This cross-sectional inter-group comparative study included 47 subjects—23 experienced and 24 non-experienced drivers. Participants completed visual search, Posner cueing, and stop-signal paradigms using PsyToolkit. Group differences were analyzed using parametric and non-parametric tests, with correlations assessing associations between driving experience and cognitive performance. Results: Experienced drivers showed a lower mean SSRT than non-experienced drivers (226.9 ± 20.1 ms vs. 237.3 ± 10.9 ms; unadjusted *p* = 0.028), with the difference reduced after Holm correction (adjusted *p* = 0.056). Mean Go reaction times were similar between groups. In the cueing task, experienced drivers showed reduced reaction times for both valid (~410 ms) and invalid (~470 ms) trials (U = 125.5, *p* = 0.001), indicating more efficient attentional reorienting. Visual Search Time was also lower in experienced than non-experienced drivers (median 950 ms vs. 1050 ms; *p* = 0.238). Conclusion: Driving experience was associated with faster inhibitory responses and better attentional efficiency, with smaller differences in visual search performance. These findings suggest experience-related cognitive adaptation and support the inclusion of attention and response-inhibition training in non-experienced driver education.

## 1. Introduction

Driving, although it seems effortless and instinctive, is one of the cognitively complex tasks [1]. It is a highly demanding activity that requires the efficient use of cognitive resources to inhibit responses when necessary to avoid accidents. Human, vehicle, road and environmental characteristics contributed to the prediction of crash injury level of non-experienced drivers. Among the various single factors, driver negligence, training and driving expertise are the most important factors affecting the crash injury level of non-experienced drivers [2]. Experience-related differences in driving behaviour are well documented, with more experienced drivers demonstrating lower fatal crash rates and improved situational awareness [3]. When driving difficulty increases, experienced drivers exhibit broader visual scanning patterns and allocate attention more efficiently, whereas non-experienced tend to focus narrowly and react more slowly to emerging hazards [4,5]. The task of driving encompasses a number of combined cognitive tasks. It involves constant intake and processing of variety of dynamic and unpredictable visual stimuli and matching these stimuli to working memory. Driving requires the coordination of multiple cognitive domains, including attention, working memory, executive function, perceptuomotor integration, and decision-making [6,7].

A driver’s ability to quickly and accurately identify and interpret relevant information from driving environment is cue and is an important factor that reduces the cognitive load of the task and allows appropriate allocation of the resources to the demands on time [8]. This skill enables rapid decision-making while reducing cognitive load, particularly in time-sensitive contexts such as driving [9,10]. It improves through accumulated task-related experiences and hence experienced drivers, based on their previous interactions in driving scenarios, identify the cues and respond to the environment more efficiently [11]. Studies have shown that experienced drivers can subconsciously activate stored cue-based knowledge, improving processing efficiency and performance under high-workload conditions [9,12].

Another key cognitive component of safe driving is response inhibition, a core executive function which refers to the ability to inhibit inappropriate or irrelevant responses [13]. In the context of driving, effective inhibitory control allows individuals to withhold automatic responses when they face sudden hazards, such as pedestrians or unexpected traffic changes. This skill matures through adolescence, with notable progress occurring by mid-adolescence and further development continuing into early adulthood [12,14]. Low executive function including response inhibition is related to impulsivity, risky driving behaviours and poor attention, especially among teen drivers [15].

Another fundamental cognitive process for the task of efficient driving is visual search. Visual search refers to the process of detecting, identifying, and interpreting objects within an image or visual environment. According to Muller in 2006, it is highly influenced by attention [16]. In driving, efficient visual search helps in hazard detection, traffic monitoring, and navigation in complex scenes [17,18]. Prior studies have shown that non-experienced drivers demonstrate restricted scanning patterns and reduced hazard perception compared with experienced drivers, particularly in demanding conditions [19,20,21]. Despite growing evidence linking individual cognitive processes to driving performance, most studies have examined these domains in isolation and have defined experience primarily in terms of years of driving, with less emphasis on real-world exposure such as driving frequency or weekly driving duration [22]. Considering that cognitive adaptation may depend not only on elapsed time but also on task repetition and intensity, examining both driving duration and exposure may provide a more nuanced understanding of experience-related cognitive changes.

However, it remains unclear whether different cognitive domains show similar or differential patterns of association with driving experience when assessed within the same group of drivers. It is also unclear whether considering regular weekly driving exposure, in addition to cumulative years of driving experience, provides a different perspective on these cognitive measures. The present study addresses this gap by examining visual search, spatial attentional orienting, and response inhibition within the same sample of experienced and non-experienced drivers, while also examining weekly driving exposure.

Given the cognitive demands of driving, this study aims to investigate the impact of driving experience on three key cognitive domains related to driving safety: cue, response inhibition and visual search. While previous studies have often examined these processes separately, the present study evaluates them within a single experimental framework. In addition, driving experience is assessed not only by years of driving but also by weekly driving exposure, providing a more comprehensive understanding of how real-world driving practice influences cognitive performance.

## 2. Materials and Methods

### 2.1. Participants

This cross-sectional inter-group comparative study included 47 healthy adults aged 20–50 years. All the subjects were included after comprehensive eye examination and had normal or corrected-to-normal vision. None of the subjects had any other ocular or systemic abnormalities. All the subjects were student, staff or drivers from SRM Medical College Hospital and Research Center, Tamil Nadu.

Participants were classified into two groups based on driving experience: experienced drivers (≥3 years of regular driving; *n* = 23) and non-experienced drivers (≤1 year of driving; *n* = 24). These experience boundaries were selected based on previous research distinguishing novice drivers with less than 1 year of driving experience from more experienced drivers with ≥3 years of experience. Accordingly, these cut-offs were used as operational definitions for the present study rather than as universally accepted definitions of driving expertise [23]. Weekly driving exposure was also recorded using self-reported driving hours and categorized as ≤5 h/week or >5 h/week to compare participants with lower and higher levels of regular driving exposure.

The study adhered to the tenets of the Declaration of Helsinki and was approved by the institutional ethics committee (Approval No: 3080/IEC/2022). Written informed consent was obtained from all participants.

### 2.2. Study Design and Objective

This cross-sectional inter-group comparative study was designed to assess differences in three cognitive domains—visual search, attentional cue, and response inhibition—between experienced and non-experienced drivers.

### 2.3. Experimental Set up and Procedure

The experiments were facilitated by PsyToolkit (v 3.7.2), a specialized web-based platform designed to expedite psychological research. This platform enabled the precise assessment of cognitive processes, whose validity had been substantiated in previous study. All tasks, including the spatial cueing, stop signal, and visual search paradigms, were meticulously configured on the PsyToolkit online platform.

**Figure 1:**
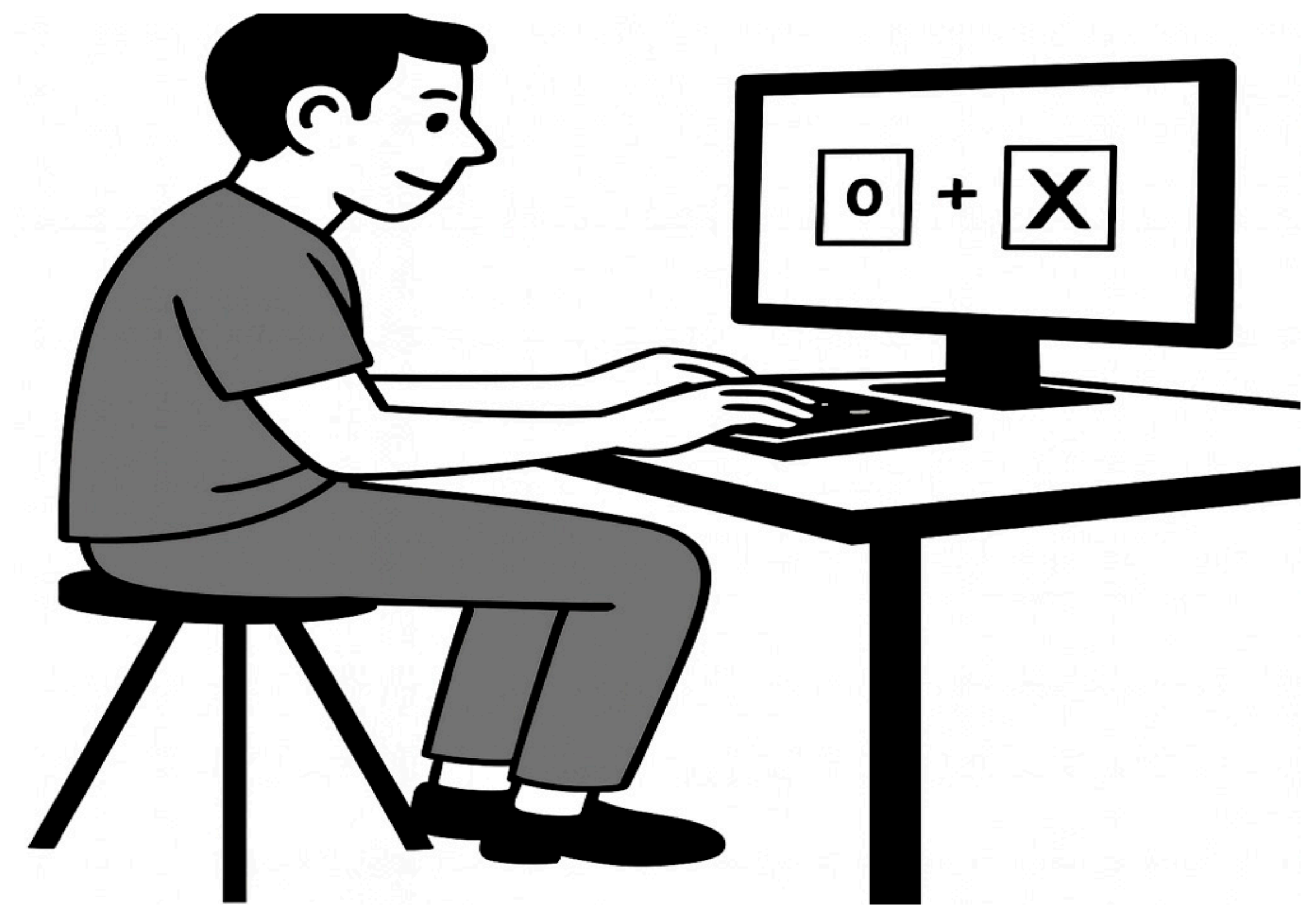
Experimental set up.

The visual stimuli were presented on a calibrated 32-inch monitor boasting a high-resolution display (1920 × 1080), which was connected to a computer optimized for precise stimulus projection. Subjects were seated comfortably at a standard distance from the monitor, and clear instructions were provided. A practice round familiarized subjects with the task, and brief breaks between tasks mitigated fatigue, which can impact reaction time and performance (Figure 1).

### 2.4. Visual Search Task

**Figure 2:**
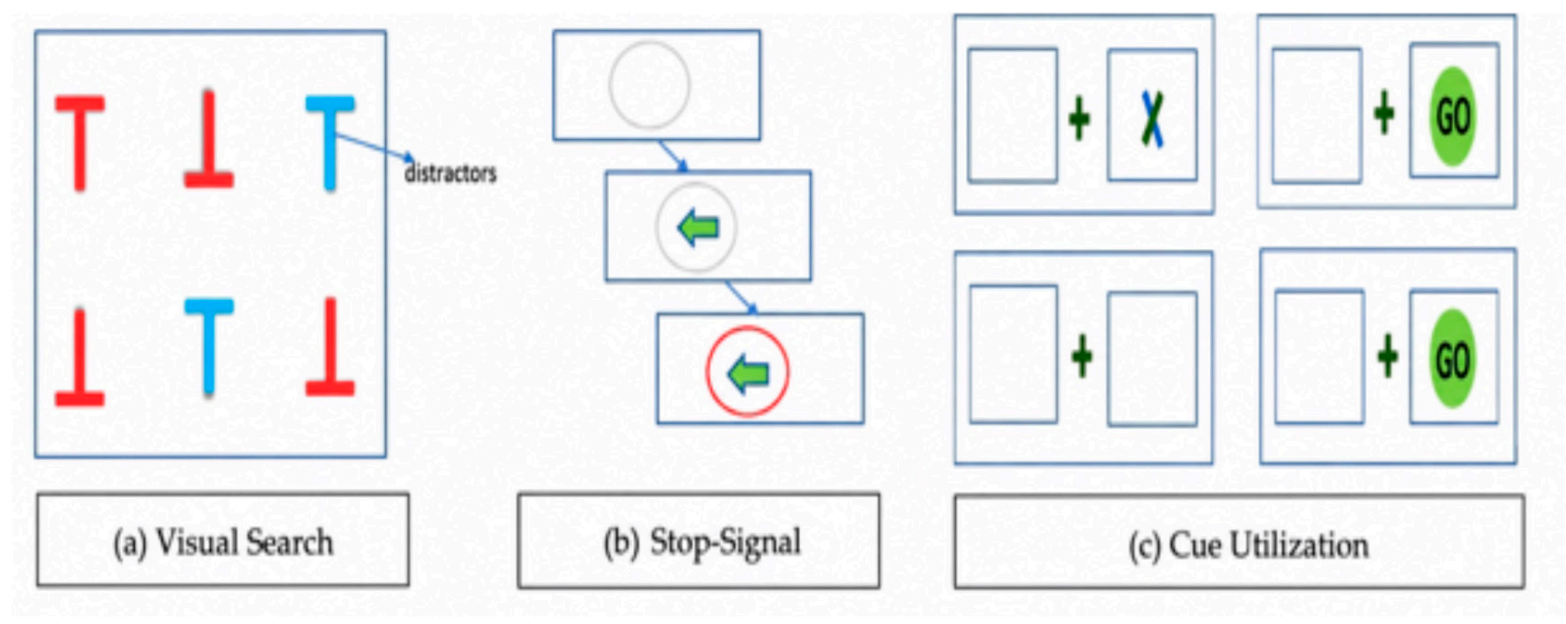
Experimental tasks used in the study: (**a**) visual search paradigm, (**b**) Stop-Signal Task, and (**c**) Posner spatial cueing task.

Visual Search Task refers to the process of actively looking for a specific target among distractors in a visual scene. A web-based Visual Search Task assessed reaction time to specific targets. Participants viewed ‘T’-shaped targets varying in orientation and colour (orange/blue) and responded to correct orientation and orange ‘T’ by pressing the space bar (Figure 2a). The task consisted of 50 search displays with 5–20 targets, examining search time as a function of target number (expected completion time: 5 min). Error messages appeared for missed targets when the subject did not respond to the target object. The primary outcome was reaction time, which is the time taken to identify stimulus among distracters.

### 2.5. Stop-Signal Task

Response inhibition was assessed using a standard stop-signal paradigm. The primary outcome measure was Stop-Signal Reaction Time (SSRT), (Figure 2b). The task consisted of 60 Go trials and 20 Stop trials, corresponding to 75% Go and 25% Stop trials.

The visual stimuli sequence for each trial consisted of a white circle initially displayed for 250 ms, followed by a fixation cross appearing in the centre of the circle for 75 ms. For “Go” trials, either a right or left green arrow appeared within the circle, displayed for 500 ms. For “Stop” trials, the white circle unexpectedly turned red after a randomly selected Stop-Signal Delay (SSD) of 100–450 ms in 50 ms increments. Thus, the SSD was randomized across trials and was not adaptively adjusted according to participants’ performance. This colour change from white to red served as the stop-signal, requiring subjects to withhold their response. The task measures subjects’ ability to inhibit prepared response. When participants successfully withheld their response following the stop signal, the trial proceeded without a response. When participants responded despite the stop signal, the response and reaction time were recorded. Reaction time, response status, and SSD were recorded for subsequent analysis. SSRT was calculated using the mean method as the difference between mean Go reaction time and mean SSD (SSRT = mean Go RT − mean SSD). The sequence of Go and Stop trials is illustrated in Figure 2b.

### 2.6. Posner Cueing Task

Attentional orienting was assessed using a Posner spatial cueing paradigm. The task incorporated both valid-cued and invalid-cued trials to assess the effects of spatial attention on response time.

All trials began with black screen displaying two yellow-bordered boxes positioned on either side of a central fixation point. The visual sequence then proceeded as follows (as shown in Figure 2c).

In valid-cue trials, an “×” appeared in one randomly selected box for 200 ms, followed by a 500 ms delay before target presentation at the cued location. In invalid-cue trials, the same spatial cue was presented, but the target subsequently appeared in the opposite box. Thus, both valid and invalid-trials contained a spatial cue, with invalid trials representing a mismatch between the cued location and the actual target location. The cueing table contained six valid-trial entries and two invalid-trial entries, corresponding to an intended 75% valid and 25% invalid trial probability. Participants completed 25 randomized trials. No separate neutral or un-cued condition was included. The target stimulus was a green circle appearing in either the left or right box. Participants responded by pressing the right shift key upon target detection. A 300 ms inter-trial interval separated consecutive trials. The primary outcome was reaction time (RT), defined as the interval between target onset and keypress.

### 2.7. Statistical Analysis

To assess the normality of our data, we employed the Kolmogorov–Smirnov test using SPSS version 21.0. Given that our dataset comprised both parametric and non-parametric variables, we utilized a combination of statistical tests. Specifically, we used independent samples *t*-tests and Mann–Whitney U tests to compare means and median distributions between groups. For continuous variables, we reported means and standard deviations. To examine the relationships between dependent and independent variables, we conducted Pearson and Spearman correlation analyses, selecting the appropriate test based on the level of measurement and distribution of the variables. A *p* value less than 0.05 was considered significant.

For the present analysis, VST, SSRT, and Posner cueing reaction time were treated as the primary cognitive outcomes, whereas analyses involving weekly driving exposure and correlation analyses were considered secondary or exploratory. A two-sided α level of 0.05 was used for statistical significance. Holm correction was applied to the primary outcome comparisons to account for multiple testing. Effect sizes and 95% confidence intervals were reported for major between-group comparisons where appropriate. Results that did not meet the predefined significance threshold were interpreted as non-significant and were not described as “significant,” “marginally significant,” or “significant trends.”

## 3. Results

Forty-seven participants were included in the analysis (23 experienced drivers, 24 non-experienced). Groups were comparable in age, gender distribution, visual acuity, and refractive error (all *p* > 0.05). Years of driving experience differed significantly between groups (*p* = 0.024) (Table 1).

### 3.1. Visual Search Task

**Figure 3:**
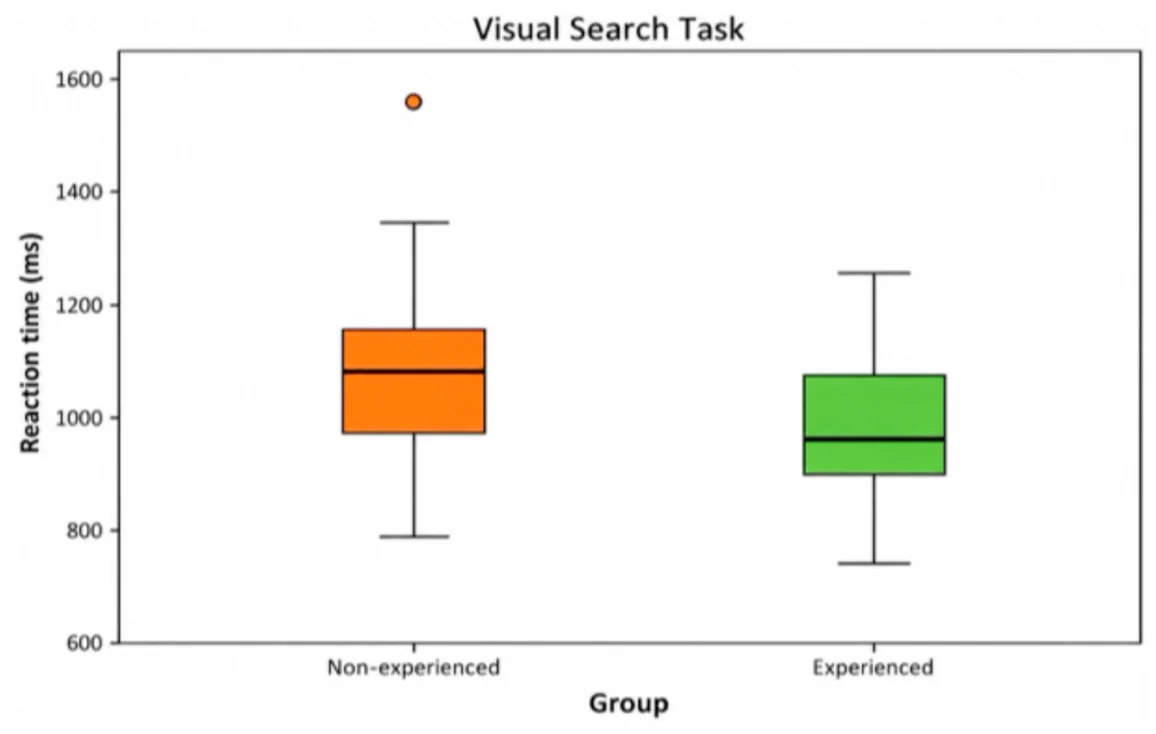
A box plot comparing Visual Search Task times (measured in milliseconds) between non-experienced and experienced groups. The colored dot represents an outlier.

Median Visual Search Time (VST) was lower in experienced drivers (950 ms, IQR 875–1050) compared with non-experienced (1050 ms, IQR 950–1200), although the difference was not statistically significant, confirmed by the Mann–Whitney test (U = 41, Z = −1.277, *p* = 0.238; Holm-adjusted *p* = 0.238). A negative association was observed between VST and driving experience, although this did not reach statistical significance (r = −0.388, *p* = 0.067) (Figure 3).

### 3.2. Stop-Signal Reaction Time

**Figure 4:**
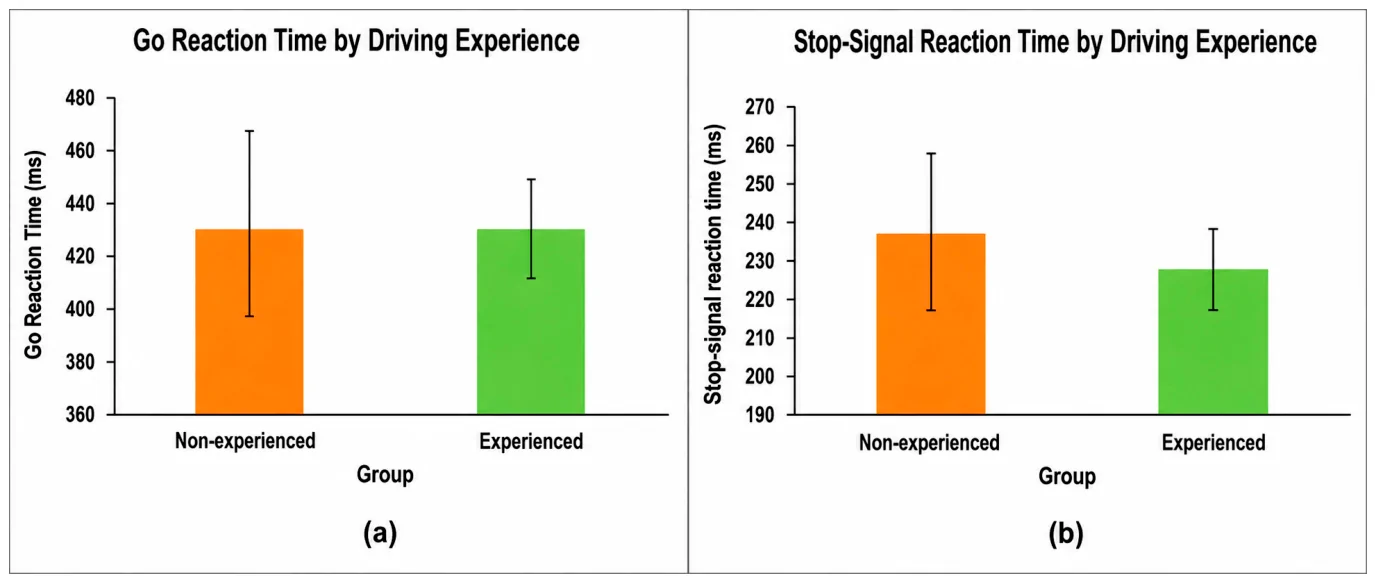
Go-reaction time and Stop-Signal Reaction Times by driving experience. (**a**) Mean Go reaction time and (**b**) mean Stop-Signal Reaction Time (SSRT) in non-experienced and experienced drivers. Error bars represent standard deviation (SD).

Mean Go reaction time did not differ significantly between experienced drivers (429.96 ms) and non-experienced drivers (432.89 ms). Experienced drivers had a lower mean Stop-Signal Reaction Time (SSRT) than non-experienced drivers (226.93 ± 20.11 ms vs. 237.25 ± 10.91 ms; t(45) = 2.20, *p* = 0.028); however, this difference did not remain statistically significant after Holm correction (adjusted *p* = 0.056). No significant difference was observed in Stop-Signal Delay between the two groups t(45) = −0.88, *p* = 0.380). The actual SSD values ranged from 165 to 290 ms in experienced drivers and from 142 to 255 ms in non-experienced drivers. Mean ± SD SSDs were 203.03 ± 26.95 ms and 195.67 ± 30.33 ms, respectively. Across participants, 940 Stop trials were analyzed, of which 391 were successfully inhibited, corresponding to an overall successful inhibition rate of 41.6%. There was no significant difference in SSD between the groups, t(45) = 0.88, *p* = 0.380 (Figure 4).

### 3.3. Posner Cueing Task

Reaction times differed significantly between groups in the cueing paradigm. Experienced drivers demonstrated shorter reaction times for both valid (~410 ms) and invalid (~470 ms) trials compared with non-experienced (~460 ms and ~570 ms, respectively); Mann–Whitney test confirms these group differences are statistically significant (U = 125.50, Z = −3.20, *p* = 0.001; Holm-adjusted *p* = 0.003). These findings indicate differences in reaction-time performance across cue conditions, but do not by themselves establish differences in attentional orienting or reorienting.

**Figure 5:**
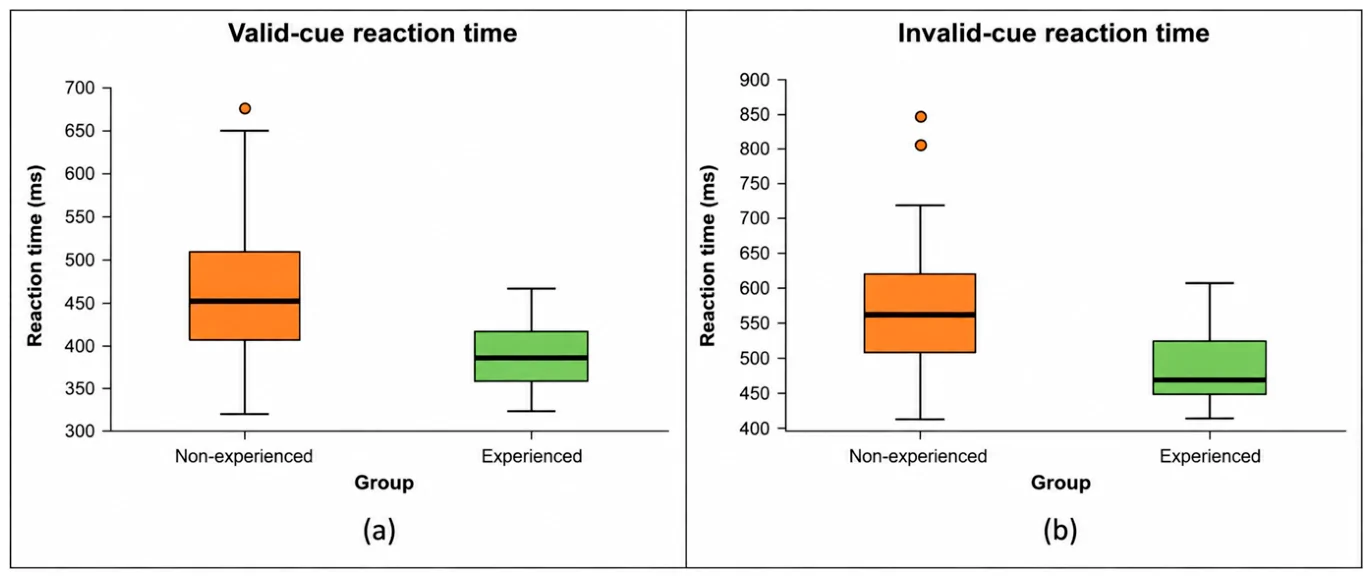
Reaction times in the Posner spatial cueing task by driving experience. (**a**) Valid-cue reaction time and (**b**) invalid-cue reaction time in non-experienced and experienced drivers.

A Spearman correlation analysis revealed that the cueing effect was more strongly correlated with invalid-cue reaction times (RTs) in both non-experienced and experienced drivers. Among non-experienced drivers, the correlation between cueing effect and invalid-cue RT was positive but not statistically significant (Spearman’s ρ = 0.383, *p* = 0.065, n = 24), while the correlation with valid-cue RT was negligible and not statistically significant (ρ = −0.014, *p* = 0.947, n = 24). Among experienced drivers, the cueing effect was significantly positively correlated with invalid-cue RT (ρ = 0.621, *p* = 0.002, n = 23), whereas its correlation with valid-cue RT was weak and not statistically significant (ρ = 0.193, *p* = 0.377, n = 23) (Figure 5).

### 3.4. Stop-Signal Task

**Figure 6:**
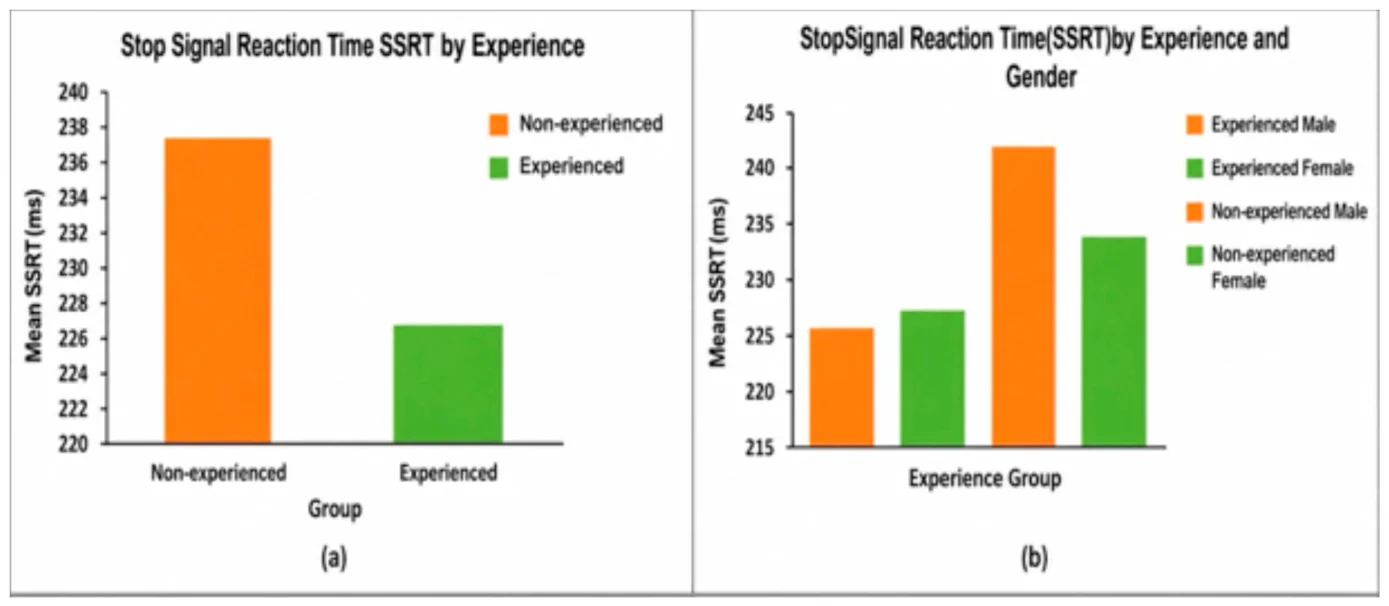
Stop-Signal Reaction Time (SSRT) by driving experience and gender. (**a**) Mean SSRT in experienced and non-experienced drivers. (**b**) Mean SSRT by gender within each driving-experience group.

Figure 6 illustrates Stop-Signal Reaction Time (SSRT) stratified by experience and gender. The no-experience group demonstrated a higher mean SSRT (237.25 ms, SE = 2.23), whereas the experienced group showed a lower mean SSRT (226.93 ms, SE = 4.19), indicating lower mean SSRT. The mean SSRT was 10.32 ms lower in the experienced group than in the no-experience group (t(45) = 2.20, *p* = 0.028).

When analyzed by gender, experienced participants exhibited faster SSRTs in both males (226.2 ms) and females (227.75 ms) compared with non-experienced males (242.4 ms) and females (234 ms). A moderate positive linear trend was observed (R^2^ = 0.445), suggesting higher SSRTs with lower driving experience, particularly among males.

### 3.5. Weekly Driving Exposure

Further analyses examined the influence of weekly driving exposure on cognitive outcomes. Drivers reporting more than 5 h of weekly driving exposure was also associated with faster performance on several cognitive measures. This finding may reflect differences in recent driving exposure; however, because weekly driving exposure is related to cumulative years of driving experience, the independent contribution of recent driving exposure cannot be determined from the present analyses.

Visual Search Task: Drivers with more than 5 h of weekly driving demonstrated faster response times across all Visual Search Tasks. Significant differences were observed in Visual Search Times (VS_5, VS_10, VS_15, and VS_20 item tasks). Mann–Whitney U tests yielded *p*-values ranging from 0.007 to 0.051 and U values ranging from 121.5 to 154.0 across the VS_5, VS_10, VS_15, and VS_20 item tasks. Some comparisons reached statistical significance, whereas the comparison with *p* = 0.051 did not meet the predefined significance threshold of *p* < 0.05.

Stop-Signal Reaction Time (SSRT) and Stop-Signal Task: There were significant differences between groups in Stop-Signal Delay (SSD) and Stop-Signal Reaction Time (SSRT), with Mann–Whitney U values of 130.5 (*p* = 0.012) and 74.5 (*p* < 0.001).

Cue and Reaction Times: Invalid cue reaction times were significantly different (U = 148.0, *p* = 0.036), while valid cue reaction times were not significant (*p* = 0.093). The cueing effect did not reach statistical significance (*p* = 0.083).

## 4. Discussion

This study investigated the association between driving experience, assessed in terms of years of driving experience and weekly driving exposure, and cognitive skills relevant to driving safety. Overall, experienced drivers demonstrated faster inhibitory responses and attentional processing, whereas differences in visual search performance were smaller and did not reach statistical significance. These findings suggest that greater driving experience is associated with differences in attentional and executive processing relevant to driving. This interpretation is consistent with skill-acquisition theories, which propose that repeated exposure to task-related environments may be associated with greater efficiency in processing relevant information and decision-making. Experienced drivers may also develop more structured mental representations of traffic environments, which could contribute to more efficient responses during dynamic driving situations [24,25]. However, because the present study used a cross-sectional inter-group design, these findings represent associations and cannot establish that driving experience directly caused the observed differences in cognitive performance.

### 4.1. Visual Search Efficiency

Though visual search differences between groups were not statistically significant, a tendency toward faster visual search performance was observed with greater driving experience. This pattern suggests that visual search efficiency may be associated with greater driving exposure, although the non-significant group difference indicates that this finding should be interpreted cautiously. Frequent driving exposure has been shown to refine visual scanning strategies and hazard detection, supporting experience-related visual processing. These findings are consistent with earlier research, which has shown that frequent exposure to dynamic driving circumstances may be associated with differences in visual scanning efficiency and hazard detection [26,27]. In practical driving contexts, efficient visual search allows drivers to detect potential hazards such as pedestrians, cyclists, or unexpected obstacles earlier, thereby providing more time to initiate appropriate responses. However, because the present study used a simplified laboratory Visual Search Task rather than dynamic road scenes, the observed Visual Search Times cannot be directly interpreted as measures of hazard detection or real-world driving safety. Even small gains in visual search efficiency can provide better safety benefits, and supports more structured, safety-focused scanning [28], but this relationship requires further investigation in more ecologically valid settings.

These findings also highlight the potential relationship between cognitive performance and task experience. Previous research has also found that better performance in attention-demanding tasks is associated with higher cue [29].

### 4.2. Inhibitory Control and Response Inhibition

The study observed significantly lower mean Stop-Signal Reaction Times (SSRTs) and Stop-Signal Delays (SSDs) among experienced drivers; however, the between-group difference did not remain statistically significant after Holm correction. This finding supports prior evidence that response inhibition is a critical cognitive function for safe driving, as it enables drivers to suppress inappropriate responses during unexpected situations [30]. Driving experience was associated with faster attentional and inhibitory responses in the present sample, with weekly driving exposure showing additional associations with selected cognitive measures.

The ability to inhibit an initiated response is particularly relevant in driving situations where rapid behavioural adjustment is required, such as braking suddenly when a pedestrian steps onto the road or when another vehicle behaves unpredictably.

The observed association between driving experience and inhibitory performance may be related to differences in driving exposure; however, further longitudinal studies are required to determine whether these differences develop as a consequence of driving experience [31].

### 4.3. Cue and Attentional Control

The Posner spatial cueing task used in the present study primarily assessed spatial attentional orienting and reorienting, which may be relevant to cue processing during driving. Significant differences in invalid-cue reaction times, including faster responses from experienced drivers, indicates differences in attentional processing when dealing with unexpected stimuli. The stronger associations between cueing effects and invalid-cue responses suggest differences in attentional reorienting in experienced individuals. Efficient reorienting of unexpected spatial information may be relevant to driving, where hazards frequently occur outside anticipated locations. This discovery is consistent with the results of [32] who highlighted the importance of attentional reorientation and contextual information processing in complex environments. Since the cueing influence was not statistically significant this may indicate that the faster responses to invalid-cue stimuli may instead reflect differences in spatial attentional reorienting.

In driving, valid and invalid cues may include changes in vehicle movement, pedestrian behaviour, or traffic flow patterns. Experienced drivers in the present sample demonstrated faster responses to invalid cue stimuli and this finding may reflect more efficient attentional reorienting and their ability to integrate them into anticipatory decision-making processes, thereby improving both efficiency and safety [33]. However, this present cross-sectional design study using Posner spatial cueing task primarily assesses laboratory-based spatial attentional orienting and reorienting and should not be interpreted as a direct measure of cue during real-world driving.

The present results suggest that experience strengthens several of these components, although the degree of improvement may vary across cognitive domains. Understanding how these processes interact may help guide the development of more comprehensive training approaches for non-experienced drivers.

### 4.4. Integration Across Cognitive Domains

The Visual Search Time (VST) and SSRT shows a significant correlation between faster Visual Search Times and shorter SSRTs suggesting an association between visual processing efficiency and executive control [34].

VST and Cueing Effect: Previous research has found that visual search and attentional orienting rely on partly different cognitive processes, which is evident from the weak connection between VST and cueing effect (SSRT).

The lack of association between SSRT and the cueing effect may suggest that these processes work separately, which is consistent with the findings of Brouwers et al. and others about the differences between inhibitory control and attentional flexibility.

These findings highlight the multifaceted nature of driving-related cognition. Safe driving requires the integration of perceptual processing, attentional orientation, and executive control mechanisms, all of which contribute to the ability to detect hazards and respond appropriately.

### 4.5. Practical Implications

The results highlight various benefits of regular driving practice and suggest several practical applications. Training non-experienced drivers with tasks that enhance visual search and inhibitory control may support the development of cognitive skills relevant to driving.

High-fidelity driving simulators could provide a platform for replicating real-world conditions thereby improving ecological validity and providing learners with greater exposure to complex driving environments.

### 4.6. Limitations and Future Directions

Several limitations have to be acknowledged. The modest sample size may have limited power to detect smaller effects, especially in visual search performance. Laboratory-based testing may not fully capture real-world driving demands, and the cross-sectional design limits causal interpretation. The Stop-Signal Task used a randomized rather than an adaptive Stop-Signal Delay (SSD) procedure. SSDs were varied across trials but were not adjusted according to participants’ inhibition performance. Consequently, the probability of successful inhibition was not controlled at a predefined level across participants. This may introduce greater variability in individual SSRT estimates than would be expected with an adaptive staircase procedure. Therefore, the SSRT findings should be interpreted within the context of the randomized-SSD design, and direct comparison with studies using adaptive SSD procedures should be made with caution. Although the groups were comparable in age, gender, visual acuity, and refractive error, other factors that may covary with driving experience, including educational background, occupation, gaming or computer experience, sleep, and fatigue, were not systematically assessed. Therefore, residual confounding by these factors cannot be excluded. Furthermore, years of driving experience and weekly driving exposure are related measures and were not examined simultaneously to determine their independent contributions to cognitive performance. Future work should incorporate longitudinal designs, larger and more diverse samples, and simulator-based or real-world paradigms to better characterize experience-related driving study. Multimodal approaches, including eye-tracking and neurophysiological measures, may further clarify the mechanisms underlying these effects.

## 5. Conclusions

This study investigated the association between driving experience, measured in terms of years of driving experience and weekly driving exposure, and cognitive skills relevant to driving safety. Overall, experienced drivers demonstrated faster inhibitory responses and attentional processing, with modest differences in visual search performance. These findings indicate that greater driving experience was associated with differences in attentional and executive processing relevant to driving. The findings align with previous research on cue, response inhibition, and visual attention, and support the development of targeted cognitive components in driver training programmes. Future studies should explore these relationships in more ecologically valid settings to better understand how experience influences real-world driving performance and safety.

However, given the cross-sectional design of the present study, these findings should be interpreted as associations and cannot establish that driving experience directly caused the observed differences in cognitive performance.

## Figures and Tables

**Table 1:** Demographic and visual characteristics of experienced and non-experienced drivers. Values are presented as median (range) where applicable.

Parameters	Experienced Drivers Median (Range)	Non-Experienced Drivers Median (Range)	*p* Value
N	23	24	NA
Age (years) *	24 (21–38)	22 (19–28)	0.715
Gender †	10:13 (43.5%/56.5%)	9:15 (37.5%/62.5%)	0.772
Visual acuity ‡ (LogMAR)	0.01 ± 0.04	0.01 ± 0.03	0.530
Refractive error (D) ‡	−0.11 ± 0.50	−0.05 ± 0.21	0.599
Driving experiences (years) ‡	4.0 ± 4.41 (3–17)	0.58 ± 0.18 (0.33–1)	0.024

* Data presented as median (range); † data presented as count (percentage); ‡ data presented as mean ± standard deviation (range where applicable); Log MAR = logarithm of the minimum angle of resolution; D = diopters; NA = not applicable.

## Data Availability

The original contributions presented in this study are included in the article. Further inquiries can be directed to the corresponding author.

## Abbreviations

The following abbreviations are used in this manuscript:

VST	Visual Search Time
SSRT	Stop-Signal Reaction Time
RT	Reaction Time
IQR	Interquartile Range
SE	Standard Error
